# Social affective forecasting and social anhedonia in schizophrenia-spectrum disorders: a daily diary study

**DOI:** 10.1038/s41537-022-00310-3

**Published:** 2022-11-14

**Authors:** Bridget Shovestul, Abhishek Saxena, Stephanie Reda, Emily Dudek, Chenwei Wu, J. Steven Lamberti, David Dodell-Feder

**Affiliations:** 1 Department of Psychology, University of Rochester, Rochester, USA; 2 Department of Rehabilitation Medicine, Mt. Sinai School of Medicine, New York City, USA; 3 School of Engineering and Applied Sciences, Harvard University, Cambridge, USA; 4 Department of Psychiatry, University of Rochester Medical Center, Rochester, USA; 5 Department of Neuroscience, University of Rochester Medical Center, Rochester, USA

**Keywords:** Schizophrenia, Human behaviour

## Abstract

Social anhedonia (SA) is a trait-like phenomenon observed across schizophrenia-spectrum disorders (SSDs). While in-the-moment social pleasure experiences are intact in SSDs, anticipatory pleasure experiences may be disrupted. Thus, the prediction of future emotions in social situations, or social affective forecasting (SAF), may play a role in SA. Therefore, we utilized daily diary methods to examine SAF in SSD and the association between SAF and SA in 34 SSD and 43 non-SSD individuals. SAF was calculated as the absolute difference between anticipatory and consummatory ratings of 13 positive and negative emotions for daily social interactions reported across eight days. Results suggest that individuals with SSDs are less accurate in forecasting negative, but not positive emotions, for future social interactions. Further, poorer forecasting accuracy of negative emotions were associated with elevated levels of SA and lower social pleasure. Together, these data suggest that inaccuracies in forecasting negative emotions may be a worthwhile intervention target for reducing SA in SSDs.

## Introduction

The capacity to experience pleasure is necessary for well-being^1^. Given what has been described by others as humans’ fundamental need to belong^2^, the consequence of experiencing pleasure is perhaps most felt in the social domain. When we are socially connected and enjoying those connections, we experience greater well-being and improved mental health^3–6^. However, when we are socially unengaged or unable to enjoy the company of others, we experience greater levels of perceived stress^7^, depression^8^, interpersonal conflict^9^, and are at greater risk for physical health problems^10^. These observations are well demonstrated by social anhedonia (SA), which is traditionally defined as the reduced capacity to experience pleasure in response to social interactions^11,12^. SA has long been characterized as a core problem in schizophrenia-spectrum disorders (SSDs)^13,14^, as well as a risk factor for the development of the SSDs^14,15^. The extent of SA in SSDs is further associated with the extent of social and occupational impairments^16–18^, exacerbation of other psychotic symptoms^9,14^, and poorer treatment outcomes^19,20^. Despite how prominently SA figures into SSDs, a critical question remains unanswered: what is the mechanism underlying SA in SSDs?

Perhaps one of the most important developments in understanding anhedonia in SSDs is a set of findings suggesting an “emotion paradox,” or a temporal component to anhedonia^21,22^. Specifically, in response to pleasant stimuli, individuals with SSDs endorse in-the-moment positive emotions^23–27^ and arousal levels^28^ comparable to that of non-SSD individuals. However, individuals with SSDs still predict future life events to be less pleasurable^24,29^ and demonstrate reduced motivation to engage in those same events^30,31^. In other words, individuals with SSDs appear to exhibit a reduction in goal-directed pleasure-based behavior, and not simply a reduced capacity to experience pleasure^22,30^. However, until recently, the emotion paradox has primarily been studied in non-social contexts, and therefore, less evidence exists in support of the emotion paradox in a social domain. Further, the few studies that do examine these processes in a social context are limited by their use of lab-based social affiliative paradigms, and thus it is still unknown whether individuals with SSDs experience anticipatory social pleasure deficits, yet intact consummatory social pleasure abilities, in response to real-world social events.

One of the more recent and compelling attempts to explain the discrepancy between estimations of current and future pleasure in SSDs draws upon cognitive-affective processing abilities. Specifically, Frost and Strauss^32^ have proposed a theory that anticipatory pleasure involves several processes^33^ including prospection, anticipatory affect, and affective forecasting (AF). Indeed, it has been argued that survival is contingent on the ability to remember emotions associated with past experiences in order to generate mental simulations of future events (i.e., prospection)^34,35^ in such a manner that brings about affect-in-the-moment (i.e., anticipatory affect). In turn, these series of events support AF, or the accurate prediction of one’s emotions during future events and motivate appropriate approach or avoidance behavior^32^. However, individuals with SSDs demonstrate difficulties encoding^36^ and recalling^37,38^ episodic memories and also exhibit working memory deficits^39^. Therefore, to the extent that memory functioning is a core ability underlying AF, we might expect unrepresentative memories of past events in SSDs to lead to negatively valanced prospections and biased anticipatory affect, which may ultimately result in low-pleasure predictions of future events, lack of motivation to pursue rewards^40–43^, and ultimately contribute to anhedonia. Thus, contemporary theories highlight the important role of AF.

AF is arguably one of the most evolved, necessary, and uniquely human capacities we possess^44,45^, guiding approach-avoidance behaviors^34,46^. In this way, individuals’ ability to accurately predict their emotions for future events directly impacts their capacity for a value-driven life^47^. Despite the significant consequences of AF inaccuracy (i.e., the pursuit of activities that yield physical/psychological harm or the avoidance of activities that would elicit pleasure), frequent errors in the prediction of future pleasure are well documented across non-clinical^45,48–50^ and, especially, SSD samples^51–54^. However, similar to the dearth of work examining the emotion paradox in a social context, until recently, AF has seldom been explored in reference to social interactions. Among the recent work examining social affective forecasting (SAF) in a SSD sample, Engel et al. ^54^ used a social ball-tossing game, Martin et al. ^53^ used a social disclosure task, and Edwards et al. ^52^ used pleasant images of individuals interacting. Indeed, laboratory-based assessments like these, and self-report measures of emotions or pleasure processes, are the primary methods used to measure SAF. However, both of these methods entail significant limitations. For instance, self-report assessments are restricted by their use of standardized, hypothetical^55^ prompts (i.e., “I get so excited the night before a major holiday I can hardly sleep”), which require participants to accurately recall their emotions during previous, similar events which have occurred during unspecified times in the past (i.e., weeks, months, and/or years prior), or, in some instances, events that participants have never experienced^33^ (i.e., “When I’m on my way to an amusement park, I can hardly wait to ride the roller coasters.”). While laboratory-based assessments of AF have advantages over self-report (i.e., less reliance on recalling past experiences), they are also limited by their inability to measure the complexities of SAF in the context of daily social behavior^22^. That is, given that emotion ratings are highly dependent on individuals’ own, unprompted enjoyment of a stimulus^52^, laboratory-based tasks are restricted to the extent they are able to measure social behavior that is meaningful and unique to each participant^22,55–57^. Between these methods, daily diary is best suited to measure anticipatory and consummatory emotions in response to real-world social company^25,26,51,58,59^ due to its ecological validity, relatively less reliance on retrospective memory, and its ability to capture temporal fluctuations in emotion ratings of the same interaction. Therefore, given the methodological limitations of previous SAF work, little is still understood regarding SAF in real-life social interactions in a SSD sample. Understanding how SAF may differ in SSDs, and the nature by which inaccuracies occur, may provide insight into the faulty processes that contribute to SAF.

In summary, previous work has demonstrated that anticipatory pleasure deficits are present in SSDs and associated with SA. Further, because a theorized core component of anticipatory pleasure is AF^32,58,60^, inaccurate affective forecasts (AFs) may contribute to SA in SSDs. Preliminary work using laboratory-based methods supports these ideas^53^. However, until now, no studies have examined AF in a social context in SSDs, using methods that capture temporal differences (reported anticipatory emotions vs. reported consummatory emotions) in emotions ratings using individuals’ real-world social interactions. Therefore, here, we examined whether SSDs exhibited differences in SAF —i.e., the accuracy with which one predicts future emotions in real-life social interactions—and whether SAF is related to SA using a daily diary method. Specifically, 34 SSD and 43 non-SSD individuals completed an 8-day daily diary questionnaire in which they reported anticipatory and, subsequently, their consummatory positive and negative emotion for daily, meaningful social interactions. We calculated participants’ SAF accuracy as the difference between their anticipatory and consummatory emotion ratings. Using these scores, we tested the following hypotheses. First, we hypothesized that the SSD group would demonstrate less accurate SAFs than the non-SSD group. Second, we explored whether emotion valence might have an impact on SAF. Although models of anticipatory emotion deficits in SSDs have historically focused on positive emotions or pleasure, recent work has argued for a broader anticipatory deficit model, which includes negative emotions^51,53,54,61^. As a result, until recently, most investigations have only assessed for positive emotion. However, existing evidence suggests that individuals with SSDs report greater anticipated negative emotion^51,53,54^ and trait-level negative emotion^17,18,62^, and exhibit less accurate forecasts of negative emotions^51,53^, relative to those without SSDs. Further, given the well documented association between elevated anticipatory negative affect and SA in SSDs^53,63^, we speculate that differences in valence might differentially relate to SAF. Finally, given the multidimensional nature of SA, it is unsurprising that a measure of SA (i.e., Revised Social Anhedonia Scale Short [RSAS^64^]) and a measure of social pleasure (i.e., Anticipatory and Consummatory Interpersonal Pleasure Scale [ACIPS^65^]) were recently found to assess different factors of social hedonism, namely, social disinterest and social reward, respectively^66^. Therefore, we included both SA measures and predicted that the extent of SAF inaccuracy would be associated with the extent of SA, and lack of social pleasure.

## Results

### Descriptive statistics

The groups were similar in most demographic characteristics and IQ (Table 1). The SSD group was moderately ill as indicated by scores on the PANSS^67^, and all were taking psychiatric medication. On the self-report measures, the SSD group reported higher levels of SA, which was a medium effect, and lower levels of interpersonal pleasure, which was approaching a large effect (Table 1). Across all participants, SA was strongly negatively correlated with interpersonal pleasure, *r*(68) = −0.81, *p* < 0.001; this association was not different between the groups, SSD *r*(31) = −0.83, *p* < 0.001, Non-SSD *r*(35) = −0.72, *p* < 0.001, 95% CI of the between-group difference = −0.33, 0.09^68^.

**Table 1:** Demographic and clinical characteristics.

Variable	Non-SSD	SSD	Group Difference
*n*	43	34	
Age, *M* (SD)	41.3 (12.5)	42.5 (12.8)	*t*(70)=.42, *p* = 0.675
Sex, *n* (%)			
Female	19 (44)	18 (53)	χ^2^(1, *N* = 77)=.29, *p* = .593
Male	24 (56)	16 (47)	
Race, *n* (%)			χ^2^(3, *N* = 75)=7.19, *p* = .066
Asian	7 (16)	0 (0)	
Black or African American	5 (12)	8 (25)	
Interracial	2 (5)	2 (6)	
White	29 (67)	22 (69)	
Not Reported	0	2	
Ethnicity, *n* (%)			χ^2^(1, *N* = 73)=.80, *p* = .371
Hispanic or Latino	1 (2)	3 (10)	
Non-Hispanic or Non-Latino	42 (98)	27 (90)	
Not Reported	0	4	
Education in Years, *M* (SD)	16.1 (2.4)	14.3 (2.6)	*t*(69)=3.12, *p* = .003
IQ, *M* (SD)	106.4 (15.1)	105.7(13.1)	*t*(74)=.19, *p* = .848
Psychotic Spectrum Disorder, *n* (%)			
Schizophrenia		16 (47)	
Schizoaffective		18 (55)	
Age of psychosis onset in Years, *M* (SD)		19.6 (7.9)	
Length of psychosis in years, *M* (SD)		23 (12.2)	
Positive and Negative Syndrome Scale, *M* (SD)			
Total		72 (19.1)	
Positive		17.8 (6.2)	
Negative		17.3 (5.7)	
General		36.9 (10)	
Revised Social Anhedonia Scale^a^, *M* (*SD*)	3.4 (3.5)	5.7 (4.8)	*t*(58)=2.24, *p* = .029, *d* = -.55, 95% CI [-1.02, -.07]
Anticipatory and Consummatory Pleasure Scale, *M* (*SD*)	83.0 (14.9)	69.7 (19.8)	*t*(60)=3.26, *p* = .002, *d* = .77, 95% CI [.31, 1.24]
Daily Diary, Estimated Marginal Mean [95% CI]^b^			
Anticipatory Positive Emotions	3.24 [3.01, 3.47]	3.14 [2.88, 3.40]	*b* = −0.10, *p* = 0.599, *R*^2^ = 0.002
Consummatory Positive Emotions	3.31 [3.06, 3.57]	3.12 [2.83, 3.41]	*b* = −0.19, *p* = 0.318, *R*^2^ = .007
Anticipatory Negative Emotions	1.18 [1.08, 1.27]	1.60 [1.49, 1.71]	*b* = 0.43, *p* < 0.001, *R*^2^ = 0.151
Consummatory Negative Emotions	1.18 [1.08, 1.28]	1.53 [1.42, 1.64]	*b* = 0.35, *p* < 0.001, *R*^2^ = 0.115

Percentages may not equal 100 due to rounding error.

^a^Non-SSD *n* = 37, SSD *n* = 32.

^b^Estimated marginal means are from linear mixed effect models with group as the predictor. Non-SSD is the reference group. Marginal *R*^2^ is reported.

On average, participants provided entries for (*M* ± SD) 5 ± 2 of the 8 daily diary days and reported a total 12 ± 8 completed, meaningful interactions across the 8 days. The groups did not differ in the number of days for which entries were provided, non-SSD = 6 ± 2, SSD *M* ± SD = 5 ± 2, IRR = 0.90, 95% CI [0.74, 1.09], *p* = 0.285, pseudo-*R*^2^ = 0.020, nor the total number of meaningful interactions reported across all days, non-SSD = 13 ± 8, SSD = 10 ± 8, IRR = 0.79, 95% CI [0.60, 1.06], *p* = 0.115, pseudo-*R*^2^ = 0.030. Both groups reported a similar number of unplanned interactions, non-SSD = 4 ± 5, SSD = 3 ± 4, IRR = 0.66, 95% CI [0.41, 1.06], *p* = 0.087, pseudo-*R*^2^ = 0.033, and planned interactions that did not occur across all days, non-SSD = 3 ± 3, SSD = 3 ± 3, IRR = 0.96, 95% CI [0.64, 1.44], *p* = 0.845, pseudo-*R*^2^ = 0.000. Descriptions of the interactions on a variety of social-affective dimensions also did not differ between groups (*R*^2^ < 0.008; Supplemental Table 1). On reported anticipatory and consummatory positive and negative emotions, compared to the non-SSD group, the SSD group exhibited higher levels of reported anticipatory and consummatory negative emotions, and no difference for reported anticipatory and consummatory positive emotions (Table 1).

### Social affective forecasting

**Fig. 1:**
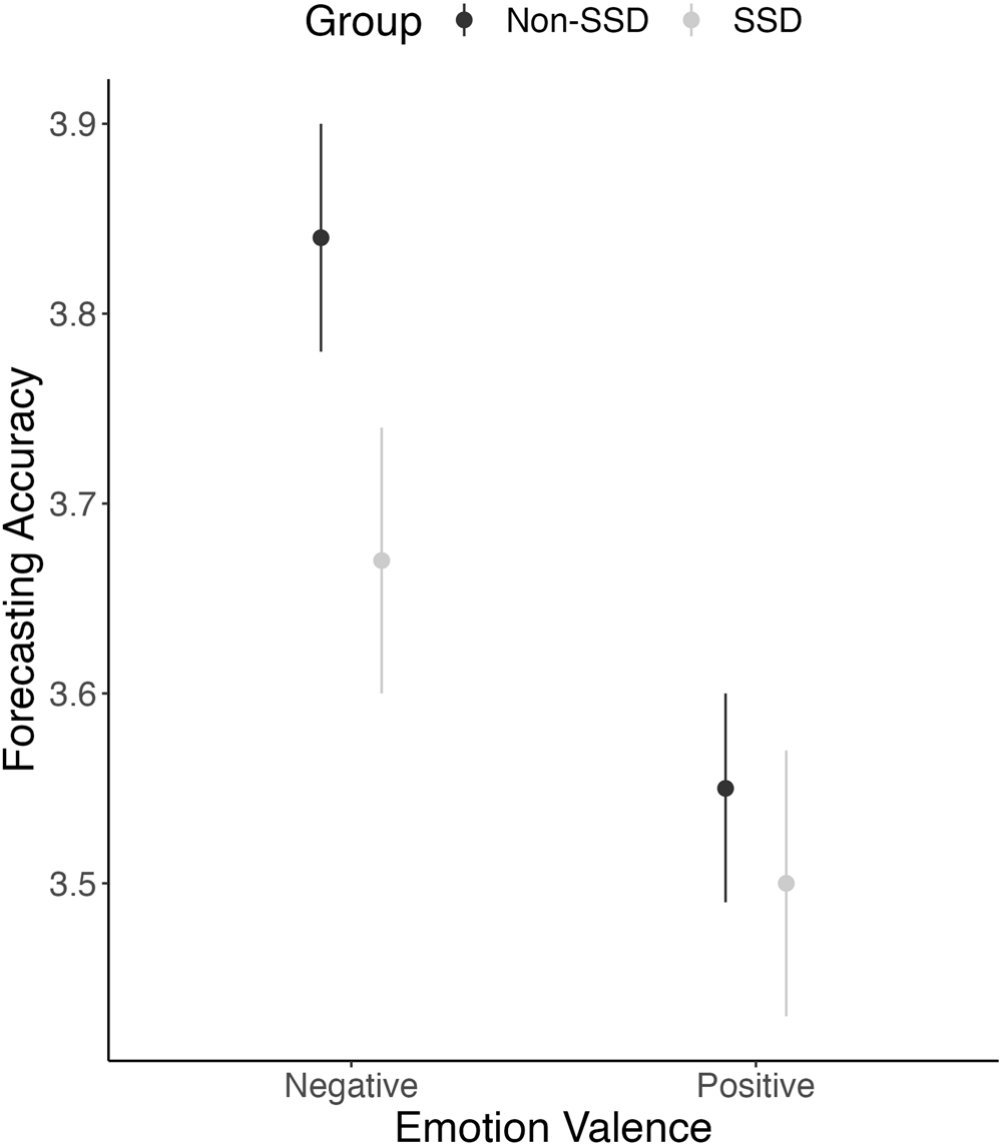
Estimated marginal means of social affective forecasting accuracy when comparing emotion valence between non-SSDs and SSDs groups. Estimated marginal means + /− 95% CI derived from linear mixed-effects models that included a random intercept for participant. Higher scores denote higher forecasting accuracy.

Our primary question concerned group differences in SAF. We evaluated the hypothesis that the SSD group would exhibit reduced SAF accuracy on an interaction-by-interaction basis. This hypothesis was confirmed. A linear mixed-effects model including a term for group and valence demonstrated an effect of group, such that on average, forecasts in the SSD group were less accurate (Table 2). There was also an effect of valence, such that on average, forecasts were more accurate for negative versus positive emotions. To evaluate whether valence impacted the nature of group differences, we conducted a follow-up model in which we included a group by emotions interaction term (Table 2), the effect of which was unexpected under the null hypothesis, and medium in size (*R*^2^ = 0.10). Post-hoc tests corrected for multiple tests revealed that the SSD group made significantly less accurate forecasts for negative emotions, compared to the non-SSD group. Group differences were not present for the forecasting of positive emotions (Table 2; Fig. 1). Both groups demonstrated greater SAF for negative versus positive emotions (Table 2).

**Table 2:** Social affective forecasting results.

Model	Term	Level	Estimated Marginal Mean [95% CI] ^a^	*b* [95% CI]	*t*	*p*	Marginal *R*^2^
Forecasting Accuracy Predicted by Group and Emotion							0.095
	Group^b^			−0.11 [−0.19, −0.03]	2.57	0.012	
		Non-SSD	3.69 [3.64, 3.75]				
		SSD	3.58 [3.52, 3.65]				
	Emotion^c^			−0.24 [−0.28, −0.21]	13.79	<0.001	
		Positive	3.51 [3.47, 3.56]				
		Negative	3.76 [3.71, 3.80]				
Forecasting Accuracy Predicted by Group by Emotion Interaction^d^							0.100
	Group * Emotion			0.12 [0.05, 0.19]	3.34	<0.001	
		^1^Non-SSD Positive	3.55 [3.49, 3.60]				
		^2^SSD Positive	3.50 [3.43, 3.57]				
		^1,3^Non-SSD Negative	3.84 [3.78, 3.90]				
		^2,3^SSD Negative	3.67 [3.60, 3.74]				

^a^Scores have been reversed, such that higher scores indicate less discrepancy between ratings and greater accuracy.

^b^Non-SSD is the reference group.

^c^Positive emotion is the reference group.

^d^Levels with a shared numerical superscript indicate that that differences in the estimated marginal means are unexpected under the null hypothesis, corrected *p* < 0.05.

**Fig. 2:**
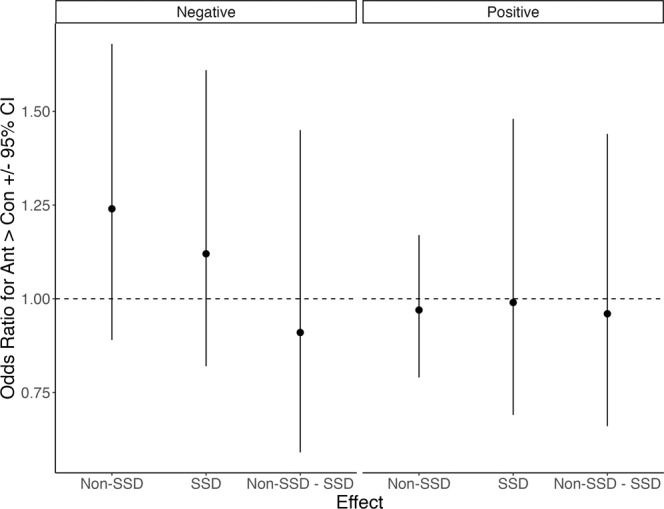
The nature of forecasting inaccuracy. Odds ratios ± 95% CI depicting the odds of rating anticipatory emotion as higher than consummatory emotion for instances when negative emotion forecasts were inaccurate (left facet) and when positive emotion forecasts were inaccurate (right facet). The Non-SSD and SSD effects represent the odds of making such a rating within each group; the Non-SSD—SSD effect depicts the odds of SSD making a higher rating for anticipatory > consummatory. All effects *p*s > 0.05.

To better understand the nature of the SAF differences for negative emotions in SSDs—that is, whether the difference was driven by higher reports of anticipatory versus consummatory negative emotions or vice-versa—we recoded each inaccurate forecast as a categorical variable indicating the direction of the inaccuracy (either over-estimation—anticipatory > consummatory—or under-estimation—anticipatory < consummatory). For inaccurate forecasts, both groups were more likely to over-estimate negative emotions, *OR* [95% CI] Non-SSD = 1.24 [0.89, 1.68], SSD = 1.12 [0.82, 1.61], but not more than what would be expected by chance (*p*s > 0.05; Fig. 2). Comparing the proportion of forecast differences between groups, being in the SSD group lowered the odds of over-estimating negative emotion by 0.91 [0.59, 1.45]; however, this difference was not unexpected under the null hypothesis, *z* = 0.44, *p* = 0.659, marginal *R*^2^ = 0.001 (Fig. 2). These findings can be taken to mean that in cases where AFs are inaccurate, the SSD group did not show a reliable pattern whereby they consistently rate anticipatory negative emotions as greater than consummatory negative emotions or vice-versa. Given the main effect of valence, such that inaccuracy was higher when forecasting positive emotions relative to forecasting negative emotions, we also explored the nature of inaccuracies for positive emotions. In contrast to inaccurate negative emotion forecasts, both groups were more likely to under-estimate positive emotions, *OR* [95% CI] Non-SSD = 0.97 [0.79, 1.17], SSD = 0.99 [0.69, 1.48], but not more than what would be expected by chance (*p*s > 0.05; Fig. 2). Comparing the proportion of forecast differences between groups, being in the SSD group lowered the odds of under-estimating by 0.96 [0.66, 1.44]; however, this difference was not unexpected under the null hypothesis, *z* = 0.21, *p* = 0.836, marginal *R*^2^ = 0.000 (Fig. 2).

Although the proportion of over- versus under-estimations were similar in the SSD group, if one form of estimation was more likely to result in worse forecasting accuracy, that could speak to the relative clinical importance of over- versus under-estimations. In line with this idea, we evaluated the association between under-/over-estimation and magnitude of SAF inaccuracy in the SSD group by regressing accuracy on interaction-by-interaction estimations (i.e., under-estimation [reference level], over-estimation) using linear mixed-effects models. For negative emotions, there was a trend-level association between estimation type and accuracy such that under-estimations (i.e., rating anticipatory negative emotion as lower than consummatory negative emotion) were associated with worse forecasting accuracy, estimated marginal mean [EMM] under-estimation=3.47 [3.37, 3.57], EMM over-estimation [95% CI] = 3.56 [3.47, 3.66], *b* = 0.09, *t* = 1.89, *p* = 0.060, *R*^2^ = 0.014. We found no such trend in the case of forecasting positive emotions, EMM under-estimation=3.39 [3.26, 3.52], EMM over-estimation=3.33 [3.20, 3.45], *b* = −0.06, *t* = 0.93, *p* = 0.351, *R*^2^ = 0.003.

### Associations between social affective forecasting, social anhedonia, and social pleasure

Finally, we evaluated whether SAF was related to SA and social pleasure. Across all participants, SA and social pleasure were related to SAF for negative emotions, and at a similar magnitude, SA marginal *R*^2^ = 0.039, social pleasure marginal *R*^2^ = 0.050, such that greater SA was related to worse forecasting accuracy, and greater social pleasure was related to greater forecasting accuracy (Table 3). These associations did not differ as a function of group. However, the robust model suggested an interaction of group and social pleasure on negative SAF, *b* = 0.004, 95% CI [0.001, 0.007], *p* = 0.025. Simple slopes analysis revealed the association between social pleasure and negative SAF was positive in both groups, with the association being more strongly positive in the SSD group, non-SSD *b* = 0.001, 95% CI [−0.002, 0.003], SSD *b* = 0.004, 95% CI [0.002, 0.007]. Neither SA nor social pleasure were associated with forecasting accuracy for positive emotions.

**Table 3:** Associations Between Social Affective Forecasting, Social Anhedonia, and Social Pleasure.

Outcome	Predictor	*b* [95% CI]	*t*	*p*	Marginal *R*^2^
Positive Affective Forecasting Accuracy					
	Social Anhedonia	−0.01 [−0.02, 0.01]	1.05	0.296	0.004
	Social Anhedonia by Group	−0.01 [−0.04, 0.02]	0.47	0.637	0.004
	Social Pleasure	0.002 [−0.001, 0.005]	1.38	0.174	0.006
	Social Pleasure by Group	0.004 [−0.002, 0.01]	1.19	0.238	0.010
Negative Affective Forecasting Accuracy					
	Social Anhedonia	−0.02 [−0.03, −0.01]	3.08	0.003*	0.044
	Social Anhedonia by Group	−0.02 [−.04, 0.01]	1.43	0.158	0.093
	Social Pleasure^a^	0.004 [.002, 0.01]	3.44	<0.001*	0.050
	Social Pleasure by Group	0.004 [−0.00, 0.010]	1.75	0.085	0.086

Asterisk (*) indicates FDR-corrected *p* < 0.05 for each group of four tests.

^a^Statistics reported reflect the non-transformed, non-robust model.

## Discussion

Accurate prediction of one’s future emotional experience is important for motivating behaviors that affect our health and well-being. One such decision impacted by AF is the choice to engage in social interactions. So important is AF on social behavior that inaccuracies may be related to SA, and its concomitants. Yet, despite decades of research on AF, little empirical evidence exists that directly address this putative association in one’s daily social life. Therefore, to help understand a potential underlying mechanism of SA, the present study is the first to use a daily diary method to address whether SAF is disrupted in SSDs and associated with SA and social pleasure.

Aside from our results on SAF, our daily diary findings converge with other literature on social interactions in SSDs, lending support to our approach. Specifically, first, we find no group differences in the number of meaningful social interactions experienced, which is supported by recent work demonstrating that clinical status does not predict frequency of social interactions^69,70^. Second, similar to non-SSDs, individuals with SSDs demonstrated similar rates of forecasting more social interactions than they engaged in and also of engaging in more social interactions than what they forecasted, which is similar to a recent finding by Merchant et al. ^71^ (although no control group was used here). At first glance, these data may be taken as evidence that SSDs do not exhibit forecasting deficits. However, unanticipated social interactions may occur and anticipated social interactions may not occur for reasons unrelated to the participant or their forecasting accuracy (e.g., participant unexpectedly runs into a close friend while grocery shopping or becomes ill and is unable to meet their parents for dinner). Third, in reference to the emotion paradox, we find individuals with SSDs report experiencing high levels of both anticipatory and consummatory negative emotions, relative to non-SSDs. In comparison, SSDs show an opposite pattern for positive emotions: Individuals with SSDs report similar levels of anticipatory and consummatory emotions relative to non-SSDs. We replicate prior work that similarly demonstrates an absence of group differences for positive emotions, yet elevated anticipatory^53^ and experienced^69^ negative emotions in those with SSDs.

These findings aside, our primary objectives were to evaluate whether individuals with SSDs exhibit deficits in SAF, whether this was impacted by valence of the emotions being forecasted, and whether SAF is associated with SA and social pleasure. In support of our first hypothesis, we find that individuals with SSDs exhibit less accurate SAF, relative to individuals with non-SSDs. Our results align with prior work showing that individuals with SSDs^53^ and schizotypal traits^63,72,73^ demonstrate inaccurate AFs, including AF for social interactions^53^. Our study extends this literature by showing that this inaccuracy occurs in a social context. One explanation for this finding comes from Frost and Strauss^32^ who argue that cognitive processing deficits in SSDs related to reinforcement learning^74^, working memory^39^ and episodic memory^37^ may be associated with inaccurate AF for future social behavior. Specifically, if prospections of future events are constructed by recalling past events^75^, then cognitive deficits may at least be partially responsible for inaccurate representations of past experiences to the extent that inappropriate, corresponding emotions are induced in the moment, which ultimately informs faulty predictions of future emotions^60^. However, future work is necessary in order to confirm that AF represents a byproduct of a series of cognitive-affective processing abilities, which might differentiate SSDs from non-SSDs.

If SAF does serve as a way in which to assess cognitive-affective functioning, then it might be tempting to assume that inaccuracies occur in the prediction of all emotions. However, consistent with findings from Martin et al. ^53^, we found that compared to non-SSDs, individuals with SSDs, make less accurate forecasts when predicting negative emotions, but not positive emotions. In other words, although SSD and non-SSD individuals demonstrate more accurate SAF of negative emotions, relative to positive emotions, group differences in SAF are only observed in reference to negative emotions, where individuals with SSDs display, on average, a greater degree of SAF inaccuracy. Together, our findings lend support for our second hypothesis that valence of emotions impacts SAF and might suggest two important conclusions. First, across the entire sample, mechanisms that govern the forecasting of positive and negative emotions are at least somewhat different. Second, the mechanisms that might differentiate negative emotion forecasting from positive emotion forecasting may help explain symptoms observed in SSDs.

When negative forecasting inaccuracies occurred in the SSD group, it was not consistently due to overestimations of negative emotions relative to the actual experience, referred to as positive incongruence errors^76^. Further, SSDs were not more likely than non-SSDs to demonstrate positive congruence errors as a source for negative forecasting inaccuracies. Together, our findings do not suggest that SAF inaccuracies in SSDs are differentially caused by an emotion paradox, or that negative emotion forecasting inaccuracies reflect a pattern by which SSDs consistently overpredict negative emotions, yet report experiencing negative emotions at similar rates to non-SSDs. Of course, as noted by Kaplan et al. ^76^, any deviation from what is predicted to what is experienced, regardless of the direction of the deviation, is detrimental; however, considering possible clinical implications, and a target for intervention, it would be useful to understand whether one type of inaccuracy—over-estimation versus under-estimation—is *more* detrimental to behavior for those with an SSD. We attempted to address this with the exploratory analysis on the type of SAF error (under- versus over-estimation) and magnitude of forecasting inaccuracy. We found a trend-level association whereby in those instances in which SSD individuals under-estimated negative emotion (versus over-estimated negative emotion), the degree of inaccuracy was greater. In other words, there may be something particularly challenging about generating accurate prospections for social situations that involve a higher degree of negative emotion. Given that this finding was trend-level and not expected, any interpretation of these findings on forecasting inaccuracy type is extremely speculative, though it may be a fruitful area for future research.

Finally, we confirmed our third hypothesis, that SAF would be associated with SA and social pleasure. Specifically, we found that SAF inaccuracy for negative emotion, but not positive emotion, was related to higher levels of SA and lower levels of social pleasure. This suggests that the consequences for inaccurate predictions about negative emotion are more consequential than those for positive emotion, even if forecasts for positive emotion are generally more inaccurate compared to negative emotion.

Taken with existing research, clinically, it may be useful to improve the accuracy of negative emotion forecasting in an attempt to ameliorate SA and increase social pleasure. Specifically, interventions aimed at helping patients challenge the overall discrepancy between anticipatory and consummatory negative emotion ratings (e.g., less under-prediction) may be instrumental in helping patients recognize how their thoughts and feelings may change in the time between anticipation and consumption^51^. Over time, the promotion of more realistic expectations of social interactions may contribute to improved decision making, which in turn can prompt fewer negative emotions, and decrease the likelihood of developing SA. Still, as previously mentioned, we did not find evidence that individuals with SSDs consistently rate anticipatory emotions as higher or lower than consummatory emotions in cases where their AFs were inaccurate. Therefore, it still remains unclear as to whether focus should be spent on challenging anticipatory versus consummatory experiences as they pertain to negative emotions. Future research will be helpful in this regard.

Several limitations are noteworthy. First, while we utilized an experiential sampling methodology (i.e., daily diary), consummatory emotions were not collected in real time and thus we are still limited by retrospective reporting. That said, the current participants’ consummatory emotions were recorded within close proximity to the emotions associated with the experienced social interaction, and not during a non-specific time in the distant past as is typical of other self-report measures. Moreover, Schneider et al. ^56^ found that across three different experience-sampling methods assessing intraindividual emotions, ecological momentary assessment (EMA) and an end-of-day diary method, similar to that used in present study, demonstrated a high correspondence (*ρ* ≥ 0.95) between measures for mean negative and positive emotion levels. Nonetheless, it would still be worthwhile testing our hypotheses using EMA methods in which participants are probed immediately following social interactions to further minimize retrospective reporting of emotions. Second, more than a third of the entire sample completed daily diary procedures during the COVID-19 pandemic when social distancing was prevalent, which may have impacted our results. Third, our sample size was modest. That said, it is similar in size to other daily diary studies in SSD samples (e.g.,^77^), and we were adequately powered to detect moderate effects. Fourth, while our data suggest that groups did not differ in the nature of their interactions, this analysis was limited by the brief descriptions of the interactions, which were generally lacking in detail. Thus, we cannot rule out the possibility that people with SSDs engage in activities characterized by lower pleasure, higher negative emotion, and/or activities in which it may be more difficult to predict one’s future affect. It would be worthwhile in future studies to use methods that allow for more precise, objective characterization of daily social interactions. Fifth, we were unable to assess whether mood state at the time of sampling might impact SAF findings. Given that negative mood states (i.e., depression) have been shown to contribute to a biased reporting of current emotions^78^ and the prediction of future emotions^79–81^, future work should explore whether mood state at the time of diary sampling impacts SAF accuracy. Finally, our data do not directly address whether SAF causes SA. Future work should utilize study designs that can demonstrate the putative causal effect of SAF on SA.

Notwithstanding these limitations, our findings broaden the scope of the literature by using a daily diary method to highlight the association between SAF and SSD, and the potential role of SAF in SA and social pleasure. We find that relative to individuals with non-SSDs, those with SSDs demonstrate greater negative emotion forecasting inaccuracies, and that the extent of this inaccuracy is related to greater SA and decreased social pleasure, regardless of diagnosis. Together, these findings may yield clinically significant implications for future interventions aimed at targeting negative SAF inaccuracies in an effort to mitigate SA and social pleasure deficits in individuals with SSDs.

## Methods

### Participants

In line with existing experience sampling studies of SSDs^24,57,59,70,77,82–84^, we aimed to recruit a sample of at least 30 participants per group. As assessed with the *EMAtools* package^85^, assuming each participant reported 8 interactions in total (1 per day), this would provide over 80% power to detect medium-sized effects, which we expected based on other studies of anticipatory and consummatory pleasure in SSDs^52–54^ and schizotypy^63^. Our final sample included 34 individuals with SSDs who met criteria for schizophrenia or schizoaffective disorder according to the Structured Clinical Interview for DSM-5 Disorders (SCID-5)^86^, and 43 non-SSD participants (Table 1), who, on average, reported a complete set (i.e., anticipatory and the corresponding consummatory emotion ratings) for 12 interactions. This gave us >80% power to detect a medium-sized effect even in the case of an ICC = 0.5.

Non-SSDs had no familial history of psychosis, any current DSM-5 psychiatric disorder, or history of psychiatric hospitalization as assessed with the SCID-5. All participants were between the ages of 18-65; fluent in English; demonstrated absence of cognitive impairment (IQ > 70); and had no history of: substance use disorder in the prior 6 months, a major neurological disorder, and head trauma or loss of consciousness. All participants were recruited via posted study advertisements in the Rochester community, including the University of Rochester’s Strong Ties Community Support Clinic, or through online study advertisement platforms (e.g., Craigslist and Research Match). Symptom severity in the SSD group was measured using the Positive and Negative Syndrome Scale (PANSS)^87^. The majority of participants (61%) came to the lab to complete self-report measures, and were then oriented to and completed daily diary procedures prior to the start of the COVID-19 pandemic in the U.S. A proportion (39%) of participants completed the study after the start of COVID-19 (i.e., participated after March 2020) and were oriented to daily diary procedures virtually via video-conferencing. However, there was no difference between groups in the number of participants who completed the study before versus during the pandemic (non-SSD *n* = 14, SSDs *n* = 14), *χ*^2^(1, *N* = 77) = 0.29, *p* = 0.588. There were also no demographic differences between participants who completed the study before versus during the pandemic, no differences in scores on the self-report measures of SA and social pleasure (see descriptions of measures below), no clinical differences between SSD participants participating in the study before versus during the pandemic, and no differences in SAF accuracy (see below). Participants were monetarily compensated for their participation in the form of cash or Amazon e-gift card after their daily diary window closed. All research procedures were approved by the University of Rochester’s Research Subject Review Board.

### Daily Diary

For the daily diary, all participants received identical instructions in which they were asked to report on all anticipated and completed meaningful social interactions. Meaningful social interactions were described to participants as social exchanges, which occurred either in person or virtually (i.e., video conferencing, texting, email, etc.), that were important to the participant or the other person/people involved, and not interactions that were non-social in nature (i.e., reading a book), those that included perfunctory and/or mechanical social exchanges, and/or involved non-humans (i.e., spending time with pets). Using their own internet-accessible device, all participants were provided a hyperlink to navigate to the electronic daily diary survey where they were asked to briefly report both the activity and the other individual(s) involved in each reported social interaction in just a few words (e.g., “getting lunch with a friend”). No participants were excluded based on the inability to access an internet-compatible device. All daily entries were monitored by members of our study team to ensure interactions fell within the boundaries of the stated parameters. The daily diary was completed over the course of eight days with participants completing their first entry on anticipatory events in the lab (prior to March 2020) or over a videoconference call via screen-sharing (following March 2020). Importantly, regardless of the format in which participants completed their first anticipatory interactions, all participants viewed the same initial daily diary interface and were guided through each step of survey with the support from a member of our study team. The following seven days, daily diary procedures were identical across formats. All participants were provided with daily email reminders to prompt the independent completion of diary daily entries each night.

For the first daily diary entry, participants were asked to provide brief descriptions of the meaningful social interactions they anticipated over the next 24 hours. Next, participants were asked how they anticipated feeling during the interaction using 13 emotions, six of which were positively valenced (enjoyment, pleasure, enthusiasm, interest, excitement, happy/joyful) and seven of which were negatively valenced (disinterest, upset, afraid/fearful, anxiety/nervousness, displeasure, anger, sadness). Emotions were rated on a 1 (*Very slightly* or *Not at all*) to 5 (*Extremely*) Likert scale. These emotions and the rating scale were adapted from the Positive and Negative Affect Schedule (PANAS)^88^, a standardized, validated measure of emotion experience. For all other daily diary entries, which were completed once per day before bed, participants were provided with the exact description of their reported anticipatory interaction from the prior day, but not their anticipatory emotion ratings, and were asked to report (1) whether they actually engaged in the interactions they anticipated having, and if they did, (2) their emotion ratings for all completed interactions based on how they actually felt during the interaction using the same adapted PANAS (i.e., consummatory emotions), and then (3) whether they experienced additional meaningful interactions they did not anticipate having. Finally, participants reported meaningful social interactions they anticipated having over the next 24 h before their next daily diary entry. While there are practical benefits to measuring reported experiences of emotions once daily, relative to multiple times per day (i.e., minimizing participant burden and subsequent attrition rates), we note that participants’ consummatory ratings are not true in-the-moment reports of experienced emotions, but instead, may be thought of as recently recalled consummatory emotions.

In line with work highlighting the importance of measuring positive and negative emotions separately^89^, using all diary entries, we averaged the individual positive emotions and then, the negative emotions, for the anticipatory ratings and, separately, if the anticipated interaction occurred, the corresponding consummatory ratings, to calculate the following scores for each interaction (min = 1; max = 5): average anticipatory positive emotions, average consummatory positive emotions, average anticipatory negative emotions, and average consummatory negative emotions. Using only scores from which anticipatory and consummatory ratings were both reported for each interaction, we then calculated SAF as the absolute difference between anticipatory and consummatory emotion ratings (min = 0, or the least possible difference between anticipatory-consummatory ratings; max = 4, or the greatest possible difference between anticipatory-consummatory ratings). This type of approach to measuring SAF, referred to as “absolute accuracy”^48^, reflects the magnitude by which participants over- or underestimate future emotion, if at all. Forecasting accuracy scores were reversed so that higher scores then represented greater congruence between anticipatory and consummatory ratings for each valence in each interaction, or greater accuracy, and lower scores indicate less correspondence, or less accuracy. We did this separately for each valence to derive a positive AF score and a negative AF score. These SAF scores were the outcome variable in the linear-mixed effects models described in additional detail below.

To assess the reliability of the daily diary positive and negative emotion constructs (and the self-report measures described below), we used the *multilevelTools* package^90^ in R^91^ to calculate between- and within-person coefficient omega^92,93^, as the data were multilevel. For positive emotions, between- and within-person omega was .99 and .93, respectively. For negative emotions, between- and within-person omega was .96 and .81, respectively.

### Self-report measures of SA and social pleasure

To examine the association between daily diary variables and SA, and social pleasure, participants completed the RSAS^64^, and the ACIPS^65^, respectively.

The RSAS is a 15-item true/false self-report questionnaire used to assess social amotivation/disinterest and lack of pleasure from social interactions. Each item is answered True (1) or False (0), and a total RSAS score is calculated as the sum of all individual items (possible range=0–15). The RSAS exhibits adequate psychometric properties^64,94,95^ and is widely used in the SA^63^, SSD^53^, and psychosis risk literature^96,97^. In our sample, omega was 0.91. Due to technical error, data was collected for *n* = 37 non-SSDs and *n* = 33 SSDs.

The ACIPS is a 17-item self-report scale that was designed to measure the capacity to experience social pleasure^98,99^. Items are scored on a 1 (*very false for me*) to 6 (*very true for me*) Likert scale (possible range=17-102). The scale demonstrates strong reliability in measuring social pleasure in community samples^100,101^, SSD samples^20^, and diverse psychiatric samples^102–104^. Previous work^105^ has shown that the ACIPS is related to, but does not completely overlap with, the RSAS. Specifically, both measures consider unique aspects of SA. Existing work suggests that the RSAS accounts for two factors: social apathy and social withdrawal^106^, while the ACIPS accounts for four factors: close/intimate interactions, group/general interactions, family-related interactions, and bonding over shared interests^99,102^. In other words, the two measures assess related, but non-overlapping aspects of social interpersonal pleasure^98^. In our sample, omega was .94.

### Data analysis

Data were analyzed in R. Demographic and self-report data were compared using Welch’s *t*-tests for continuous data and chi square tests for categorical data. Effect sizes for continuous data are reported as Cohen’s *d* with 95% CIs. Count data from the daily diary—number of days completed, number of interactions reported, number of anticipated interactions that did not occur, number of unanticipated interactions—were compared between groups with Poisson regression or, when data were overdispersed, negative binomial regression^107^. We report the incident rate ratios (*IRR*) and pseudo-*R*^2^ as described in Coxe et al. ^107^ All participants had at least one complete set of entries (i.e., anticipatory and the corresponding consummatory ratings) for the same interaction. We explored whether the nature of the social interactions differed between groups by submitting participants’ descriptions of their interactions to text analysis with Linguistic Inquiry and Word Count (LIWC) software^108^. Using LIWC, we derived the proportion of words that fell within 10 semantic categories of interest that indexed social-affective information (positive emotion, negative emotion, social, family, friend, affiliation, achievement, power, reward, risk). As these data were multilevel with interactions nested within participant, we analyzed these data with linear mixed-effects models including a random intercept for participant. We note that participants were asked to keep their descriptions brief and the mean number of words per description was small, *M* ± *SD* = 4.3 ± 2.9, meaning that there was little variance in many of the LIWC categories. Thus, these exploratory analyses should be interpreted with caution.

As all other daily diary data had a similar multilevel structure with interactions nested within participant, we used similar linear mixed-effects models for continuous outcomes and logistic mixed-effects models for categorical outcomes as described above. We report unstandardized *b* values or *ORs* along with their 95% CIs, and marginal *R*^2^ based on Nakagawa and Cuthill^109^. We tested these models using the *lme4*^110^, *lmerTest*^111^, and *performance*^112^ packages, and extracted estimated marginal means and performed post-hoc tests using the *emmeans* package^113^.

To test the effect of group on anticipatory and consummatory negative and positive emotions, we conducted four linear mixed effects models with group as the predictor. For the SAF analysis, we first tested a model to evaluate the effect of group and valence on forecasting accuracy. Next, we tested whether group differences changed as a function of valence by including a group by valence interaction term in the model. Post-hoc tests were corrected for multiple comparisons.

Anticipatory and consummatory negative emotion scores from the daily diary were positively skewed (skew = 2.21), and SAF scores were negatively skewed (positive emotions skew = −1.74; negative emotions skew = −2.24). We dealt with this in two ways, first, by re-running models with transformed data (log transformed for positively skewed data and Tukey-transformed for negatively skewed data) and second, by re-running models with robust linear mixed-effects regression using the *robustlmm* package^114^. As findings were unchanged, we report data from the non-transformed, non-robust models.

To evaluate the association between SA, social pleasure, and forecasting accuracy, we conducted separate linear mixed-effects models with either positive or negative AF as the outcome, and either RSAS or ACIPS score as the predictor, and the interaction between group and RSAS/ACIPS, resulting in four tests per forecasting accuracy outcome. We consider findings to be unexpected under the null hypothesis when *p* < 0.05 after FDR-correction for each set of four tests. Skewed data were handled in the same manner as described above. Only one finding changed after dealing with the data skew—the interaction between group and ACIPS in predicting negative SAF—which is reported in the Results.

## Supplementary information

Supplemental Material

## Data Availability

The datasets analyzed in the current study are available on the Open Science Framework, https://osf.io/8ja9v/?view_only=80911ef0669e484e9542fda12571a53c.
