# Supplementary material for: Social affective forecasting and social anhedonia in schizophrenia-spectrum disorders: a daily diary study
Source: Schizophrenia (Heidelb). 2022 Nov 14;8(1):97. doi: 10.1038/s41537-022-00310-3 (PMC9663197; doi:10.1038/s41537-022-00310-3)
Supplement: Supplementary file 1 — Supplemental Material [file 41537_2022_310_MOESM1_ESM.docx]

**Supplemental Table 1**

*Group Differences in Descriptions of the Social Interactions*

| LIWC Category | Non-SSD, Estimated Marginal Mean [95% CI] | SSD, Estimated Marginal Mean [95% CI] | Group Difference |
| --- | --- | --- | --- |
| Positive Emotion | .79 [-.02, 1.60] | 1.49 [.49, 2.49] | *b*=.70, *p*=.287, *R*^2^=.005 |
| Negative Emotion | .15 [-.07, .38] | .00 [-.28, .28] | *b*=-.15 *p*=.394, *R*^2^=.006 |
| Social | 32.4 [28.2, 36.6] | 29.9 [24.7, 35.1] | *b*=-2.52 *p*=.455, *R*^2^=.003 |
| Family | 10.57 [7.68, 13.50] | 8.05 [4.53, 11.60] | *b*=-2.52 *p*=.272, *R*^2^=.007 |
| Friend | 5.41 [3.22, 7.61] | 4.52 [1.85, 7.20] | *b*=-.89 *p*=.609, *R*^2^=.001 |
| Affiliation | 16.0 [12.3, 19.6] | 14.6 [10.2, 19.0] | *b*=-1.35 *p*=.639, *R*^2^=.001 |
| Achievement | 1.79 [.78, 2.80] | 2.03 [.78, 3.28] | *b*=.25 *p*=.761, *R*^2^=.000 |
| Power | 2.90 [1.32, 4.47] | 3.29 [1.35, 5.22] | *b*=.39 *p*=.756, *R*^2^=.000 |
| Reward | .59 [.18, 1.00] | .75 [.24, 1.27] | *b*=.16 *p*=.631, *R*^2^=.000 |
| Risk | .05 [-.03, .12] | .01 [-.08, .10] | *b*=-.04 *p*=.543, *R*^2^=.001 |

*Note*. Estimated marginal means are derived from linear mixed-effects models with a random intercept included for participant. Values represent proportion of words falling within the LIWC category. Non-SSD is the reference group.
